# A Breakthrough in R&D for Neglected Diseases: New Ways to Get the Drugs We Need

**DOI:** 10.1371/journal.pmed.0020302

**Published:** 2005-09-08

**Authors:** Mary Moran

## Abstract

New research suggests that long-held beliefs on neglected disease drug development activity are no longer accurate.

Research suggests that long-held beliefs on neglected-disease drug development activity are no longer accurate, and that these inaccurate beliefs have led—and are leading—to poorly designed and targeted government policies and incentives. On a more positive note, this research also highlights opportunities for better-targeted government policies that will more closely match the reality of neglected-disease drug development and the needs of public and industry groups.

## Current Perceptions

Current government policies to stimulate development of new drugs for neglected diseases are based on a set of shared understandings.

One of these understandings is that only 13 new drugs have been developed for neglected tropical diseases since 1975, with the main problem being that these diseases are simply non-commercial for companies to invest in [1]. Another is that, although public-private partnerships (PPPs) for drug development have started, they are problematic. (In this article, PPPs are defined as public-health-driven not-for-profit organisations that drive neglected-disease drug development in conjunction with industry groups.) Governments are uncertain which of the plethora of PPPs they should support, particularly as most are thought to be too young to judge their success. At the time this article went to press, the Initiative on Public-Private Partnerships for Health (http://www.ippph.org/) listed 92 PPPs in its database (this number includes all PPP activity, including the small number of organisations that make drugs, vaccines, and microbicides; and one-off partnerings such as donations and cut-price deals).

The general view is that PPPs are inexperienced in drug development, and may eat up public cash without delivering the tools we need, while the real experience and capability in drug development lies with multinational pharmaceutical companies, which must be brought back into the neglected-disease field if we are to achieve success. For example, the Commission for Africa recently stated that we need to increase neglected-disease R&D by “giving large pharmaceutical firms incentives to investigate the diseases that affect Africa, instead of focusing on the diseases of rich countries” [2].

The logical outcome of these beliefs is to focus on new policies to commercialise neglected-disease markets for large companies, which seek peak sales of around $500 million per year to justify investment. This commercialisation approach is generally based on supplementing low developing country purchasing power with large—usually billion-dollar—market “pull” incentives, such as transferable intellectual property rights or advanced purchase commitments underwritten by Western governments.

The above all sounds fairly sensible. The only problem is that most of the above statements no longer hold true in the post-2000 world of neglected-disease drug development, and, as a consequence, government policies based on these beliefs are at risk of missing the mark.

## The 2005 Reality

The landscape of neglected-disease drug development has changed dramatically during the past five years (I am not discussing vaccines, only drugs). There were 63 neglected-disease drug projects under way at the end of 2004, including two new drugs in registration stage and 18 new products in clinical trials, half of which were already at Phase III. Assuming there is sufficient funding, at standard attrition rates these projects would be expected to deliver eight to nine new neglected-disease drugs within the next five years, even if no further projects were commenced after this time (Figure 1). This expected yield was calculated using attrition rates from the Tufts Center for the Study of Drug Development [3] combined with PPP-specific rates when available. New projects have been commenced since the end of 2004, and activity is expected to further increase as newer PPPs and institutes get into stride.

**Figure 1:**
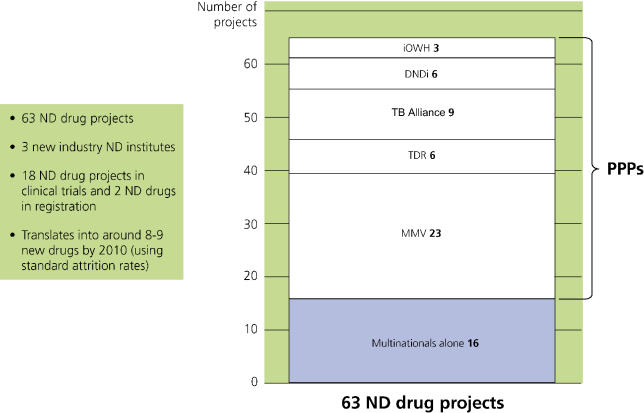
Active Neglected-Disease Drug Projects by Institution* (Dec 2004) DNDi, Drugs for Neglected Diseases Initiative; IOWH, Institute for OneWorld Health; MMV, Medicines for Malaria Venture; ND, neglected diseases.

This increase in activity is not a passing trend, but is a sign of deep-seated structural changes. In particular, it reflects the formation since 2000 of new pharmaceutical industry neglected-disease institutes, now employing over 200 scientists, and the creation of new drug development PPPs, which now conduct three-quarters of all identified neglected-disease drug development, sometimes in partnership with these industry institutes.

This renewed activity—at a level unheard of in the past two decades—commenced largely in the *absence* of significant new government incentives and generally without public intervention. Eighty percent of PPP drug development activity is funded through private philanthropy, while the industry institutes are largely self-funding, although sometimes with PPP project funding input. (One industry institute did benefit from generous support from the Government of Singapore.)

These findings led the Pharmaceutical R&D Policy Project at the London School of Economics, a project funded by the Wellcome Trust, to a closer examination: where is this new activity centred, what is motivating it, and what has suddenly made this “non-commercial” R&D possible? In line with its mandate, the project also sought to assess the performance of the different actors, in terms of both health outcomes and cost-efficiency. Our findings are set out below.

## Neglected-Disease R&D Activity

**Multinational drug companies** now conduct half of new neglected-disease drug development activity (32 projects), either working with PPPs or working alone (usually with a view to subsequent partnering). In all cases, these companies are working on a *non-commercial basis*—that is, they are not motivated by commercial returns in neglected-disease markets—and they have agreed to provide the final products to poor patients in developing countries at not-for-profit prices. The bulk of this activity is accounted for by the four companies that have formal neglected-disease divisions—GlaxoSmithKline, Novartis, AstraZeneca, and Sanofi-Aventis—while four other companies have less formal neglected-disease activity, conducting perhaps one or two projects each, and generally on a more serendipitous basis.

A further half of the identified 60-plus neglected-disease drug projects are conducted by **smaller-scale commercial firms** working with PPPs, including small pharmaceutical companies, contract research organisations and developing country firms, and academic drug developers. The non-academic part of this activity is on a purely *commercial* basis, with small firms working on a different scale and being motivated by far smaller commercial returns than large multinational pharmaceutical companies. PPPs now spend as much on small company R&D as they do on multinational company projects. The R&D drug landscape for neglected diseases is shown in Figure 2.

**Figure 2:**
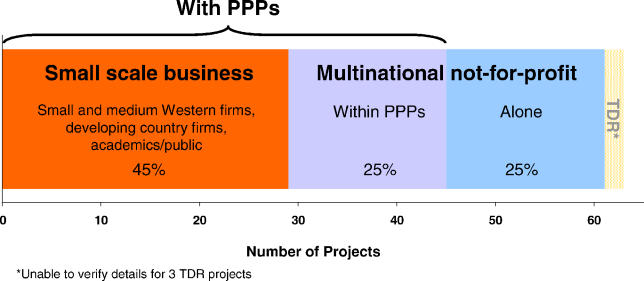
The R&D Drug Landscape for Neglected Diseases (Dec 2004)

Three-quarters of all identified R&D (47 projects) was conducted by **drug development PPPs**, often working with the large and small pharmaceutical companies mentioned above. Nearly one-third of these projects are at the clinical trial stage, including seven drugs now in Phase III trials, and two further products are in the registration stage—rectal artesunate by the Special Programme for Research and Training in Tropical Diseases (TDR) (http://www.who.int/tdr), and paromomycin by the Institute for OneWorld Health (http://www.iowh.org) and TDR. Based on standard attrition rates, these PPP portfolios would be expected to yield six to seven new neglected-disease drugs within five years. This is a high proportion of the total eight to nine new drugs expected from all current avenues.

Once these data became clear, we were faced with the question of why pharmaceutical companies were investing in neglected-disease drug development. Current thinking—and our own initial beliefs—told us this was impossible without new commercial incentives, and no such incentives had been introduced.

## Motivations and Facilitating Factors

An examination of the 60-plus neglected-disease projects now under way showed that our initial understanding of company motivations and PPPs' role was incorrect. In particular, commercial incentives were largely irrelevant to the decision by multinational companies to re-enter the neglected-disease field, while small companies involved in neglected-disease R&D were indeed responding to existing commercial drivers. In most cases, the involvement or planned involvement of PPPs was crucial to company activity, commercial or otherwise.

### 

#### Multinational companies.

Big companies involved in neglected-disease R&D were not motivated by commercial returns in the neglected-disease market, but rather by longer-term business considerations, including: (1) minimising the risk to their reputation stemming from growing public pressure on companies over their failure to address developing country needs; (2) corporate social responsibility and ethical concerns; and (3) strategic considerations (for example, positioning themselves in emerging developing country markets, or building access to low-cost, high-skilled developing country researchers).

This renewed neglected-disease activity has been made possible by a major structural change in the way multinational companies approach neglected-disease R&D. Instead of conducting a limited number of more expensive late-stage drug development projects (the pre-2000 model), companies have moved *upstream* to the less expensive and more innovative drug discovery stages—allowing them to control costs and resource inputs to levels more acceptable to shareholders. The resulting drug leads can then be developed in conjunction with public partners. These public partners (usually PPPs) facilitate further development by subsidising clinical trial costs; by providing the necessary public health skills and developing country knowledge for clinical trials, registration, and implementation; and by sharing the risk of trials in important but high-liability patient groups, such as children and pregnant women.

This partnering model, sometimes called the “no profit–no loss model”, allows companies to participate in neglected-disease research (often providing substantial in-kind inputs) while still protecting shareholder value, and manufacturing and distributing final products to developing country patients at no mark-up. This has three clear advantages. First, it provides a source of high-quality innovative industry drug leads. Second, it uses the public health sector in its area of maximum comparative advantage (developing country clinical trials rather than drug design). Third, it provides final products to poor patients at not-for-profit prices.

#### Small companies

Small companies, on the other hand, have largely commercial motivations. Some see neglected-disease markets, particularly larger markets such as tuberculosis (TB) and malaria, as sufficiently attractive to warrant investment and will pursue these even without public support. For example, Zentaris (the small company that developed and registered the new anti-leishmaniasis drug, miltefosine), noted that: “While such a market would be negligible for a big pharmaceutical company, it has a good economic scale for us” (personal communication). A second—and potentially much larger—category is that of small firms that can use “add-on” neglected-disease R&D to promote their Western commercial goals. Examples of such goals are to expand information on their core commercial compounds, or help to establish proof-of-concept for a technology that can subsequently be transferred to commercial markets. These firms generally seek and need substantial PPP support, including full cost coverage and significant skills input—and are unlikely to continue neglected-disease R&D for developing countries if this support is not forthcoming. Finally, commercial contract research organisations increasingly see neglected-disease R&D as an interesting niche sector, and are now involved on a commercial basis in one-third of PPP projects.

While promising, small company neglected-disease activity remains largely under-exploited. Most small companies continue to be deterred by the substantial barriers to entry that large, disseminated, and unfamiliar developing country markets pose, while firms with a primary Western focus can have difficulty concluding financial agreements with cash-strapped PPPs, particularly if their intellectual property concerns are not adequately addressed.

#### PPPs

As noted above, the presence of PPPs is probably essential to multinational company participation, and plays a catalytic role in encouraging small Western-focussed companies to expand their remit to neglected-disease indications. However, PPPs also serve other useful functions, particularly from the public perspective, including: (1) integrating and coordinating multiple industry and academic partners and contractors along the drug development pipeline; (2) allocating public and philanthropic funds to the “right” kinds of R&D projects from a public health perspective (for example, since 2000, two-thirds of PPP R&D spending has gone directly to industry, almost equally divided between large and small companies, while a further one-third has been allocated to academics to translate basic research into new drug leads); and (3) managing neglected-disease drug portfolios, including selection and termination of projects based on their relative merits.

By virtue of these functions, PPPs are stimulating alternative approaches in addition to “classical” one-to-one partnerships with multinational pharmaceutical companies (these now represent only about one-third of all PPP projects). Their coordinating and integrating role allows PPPs to develop compounds from many different sources even if there is no interested industry partner. For example, the TB Alliance manages and integrates development of PA-824 (a Chiron compound that the company itself did not want to pursue through the full R&D process) on a purely subcontracted basis and without formal development partners.

Alternatively, by pairing up small Western companies or academics with developing country manufacturers, PPPs can—and do—foster a neglected-disease drug development pipeline that is far cheaper than the traditional commercial approach (see “Cost-efficiency” section). Nearly one-quarter of current PPP projects involve developing country pharmaceutical firms as the manufacturing (and sometimes development) partner for a range of compounds from academia, the public domain, or small firms. For example, the Medicine for Malaria Venture's synthetic peroxide project brings together academic discovery and early-development partners (Uni Nebraska, Uni Monash, Swiss Tropical Institute) with Ranbaxy (India) as the development and trial manufacturing partner.

## Performance of the Different Approaches

Although increased neglected-disease R&D is always welcome, it is also important that this R&D is targeted to optimal health outcomes for developing country patients, and that it represents the most cost-efficient use of public and philanthropic funding (that is, that patients see maximum health returns for every dollar spent).

In order to assess performance of the various actors, we devised a range of metrics, including health value of the final products (safety, efficacy, suitability, and affordability for developing country patients), level of innovation, capacity, drug development times, cost-efficiency, and cost.

Measurement of the various drug development approaches against these metrics showed that both industry working alone and public groups working alone performed more poorly on most metrics than public–private collaborations. In other words—and perhaps unsurprisingly when we consider the matter more closely—drug development for neglected diseases is optimised by combining industry drug development expertise with public neglected-disease expertise. Below is a summary of outcomes against a sample of metrics.

### 

#### Health outcomes

The PPP approach delivered the best health outcomes for developing country patients. Eight neglected-disease projects (Artemotil, Paluther, Coartem tablets paediatric label extension, Lapdap, Biltricide, Impavido, Ornidyl, and Mectizan) were conducted in public-industry collaborations (with TDR). Three of the resulting products had a major impact on developing country health—Mectizan (ivermectin), which halved the global burden of onchocerciasis between 1990 and 2000 [4]; praziquantel, which has helped to control schistosomiasis in Brazil, the Mahgreb, the Middle East, China, and the Philippines [5]; and the TDR-assisted label extension of Coartem tablets for paediatric use, which has delivered Africa its first safe, effective, suitable new anti-malarial for many years. We note that praziquantel's impact was greatly increased by the advent of a cheaper, simpler generic formulation by Shin Poong, which allowed broader rollout than the originator product, Biltricide.

By contrast, virtually all of the 13 drugs developed by industry working alone had a low *overall* health value for developing country patients (although, as noted above, industry has now largely moved to a partnering approach), with only one drug being widely accessible and useful in the developing world (Zentel/albendazole). These 13 drugs were Zentel, Lariam, Malarone, Mycobutin, Paser, AmBisome, Arsumax, Coartem original registration for adults and children above 10 kg in a four-dose (not six-dose) formulation, Halfan, Priftin, Rifampin, Rochagan, and Vansil. The chief obstacles to wider developing country use of industry-alone drugs were their high price—often due to expensive active pharmaceutical ingredients or high formulation cost—and their poor suitability to the target populations. Examples include the development of intravenous formulations suited to Western patients but difficult in poor-country settings, and exclusion of important patient groups, such as children with malaria or HIV-positive patients with TB.

#### Level of innovation and speed of development

Incremental innovation can offer marked benefits to patients. For instance, fixed-dose combinations of existing drugs can greatly improve ease of use and compliance; follow-on drugs in the same class may improve safety and efficacy; and paediatric formulations can make childhood treatments simpler and more reliable. However, if we are to effectively manage health outcomes in the long-term then we must also overcome drug resistance, which is a growing problem for many neglected diseases, including malaria, TB, leishmaniasis, and sleeping sickness. To do so, we need to focus R&D on “breakthrough” innovation—that is, novel compounds with a novel mechanism of action against parasites and microbes—as well as on activities that simplify or improve existing therapies from the patient perspective. Measurement of the level of “breakthrough” innovation under each approach shows that PPPs and industry partnering approaches perform best. Nearly half of all PPP projects (49%) and more than half of industry partnering projects (63%) are in the “breakthrough” category, compared to only 8% of drugs developed by industry working alone under the pre-2000 model.

The 8% number should not, however, be compared directly with the post-2000 number since the former is based on registered drugs while the latter is for a portfolio of ongoing projects. Given that R&D of “breakthrough” drugs is associated with higher attrition rates, the profile of finished drugs coming out of the post-2000 portfolio is likely to include fewer innovative products. Attrition rates alone, however, cannot account for the much higher share of breakthrough innovation post-2000. The key explanation for this difference is the recent major shift in industry neglected-disease R&D strategy, as noted above, where the serendipitous approach that characterised the past 25 years has given way to one that is specifically focussed toward “breakthrough” innovation. In the long-term, this approach will only deliver high-innovation products, irrespective of attrition rates.

Although the level of innovation is important, it is equally important that innovative R&D projects move quickly to bring new drugs to patients who need them. Time metrics show that PPP drug development trajectories matched or exceeded industry standards (based on data from [3] and [6]). In particular, they were significantly faster than public-alone drug development (see Figures 3 and 4); and they generally exceeded industry trajectories for neglected-disease new chemical entities (although the latter are too few in number to draw significant conclusions).

**Figure 3:**
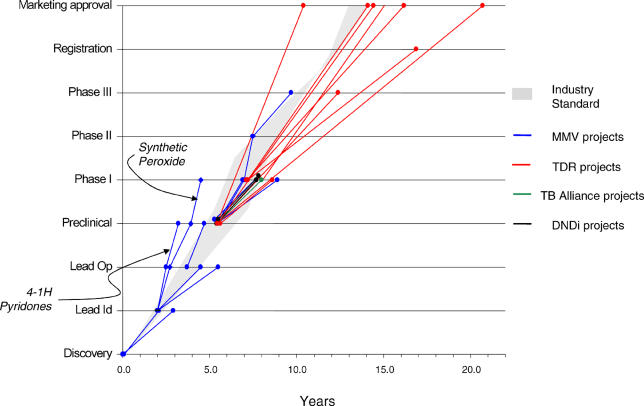
PPP Timelines DNDi, Drugs for Neglected Diseases Initiative; MMV, Medicines for Malaria Venture.

**Figure 4:**
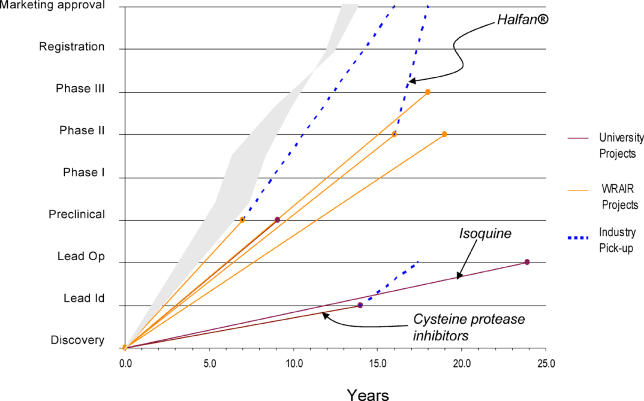
Public Timelines WRAIR, Walter Reed Army Institute of Research.

#### Cost-efficiency

The overall cost-efficiency of PPPs was superior to other approaches—partly, but not only, due to their ability to leverage substantial in-kind inputs from partners and by the exclusion of costs of capital from the PPP “push” model. The total cost of collective PPP drug development activity from 2000 to 2004 (excluding TDR, for which numbers were not available) was $112 million, including cost of failure for more than 40 projects (ten of these in clinical trials, including four at Phase III). Confidentiality agreements prevent us from disclosing project costs in many cases, in particular when company partnerships are involved. Full per-project costs were more readily disclosed on projects involving academics, developing country firms, and paid sub-contractors, and were remarkably low in most of the cases we examined. For example, Medicines for Malaria Venture's synthetic peroxide project has moved from exploratory, through lead identification, lead optimisation, and pre-clinical, and into Phase I trials at a total cost of $11.5 million. (Costs of completed projects will, of course, be higher.)

The industry cost of developing a new chemical entity for Western markets is substantially higher, estimated by the Tufts Institute at $802 million per drug including cost of failure and cost of capital, and at $403 million for out-of-pocket R&D costs per drug (including cost of failure) [3]. While indicative, these numbers do not hold fully for neglected-disease drug development, which some companies suggested at interview would be substantially cheaper due to lower developing-country trial costs (for example, around $50 million to take a new compound from discovery through to the start of clinical trials). Even using these lower estimates, however, figures to date suggest that PPPs can still be expected to perform significantly better on cost-efficiency and cost.

#### Overall performance

It is important to note that these outcomes are not evidence of the capacity of the individual players, but rather of the ability of different R&D approaches to deliver optimal outcomes. A company working in a partnership may be able to deliver a better outcome than the same company working alone, for a number of reasons. Companies working alone (as was generally the case under the pre-2000 model) tend to reduce the cost and risk of neglected-disease drug development by focussing on less-expensive, less-risky, “adaptive” R&D such as label extensions of veterinary drugs to humans, or new formulations of existing drugs, and/or by working slowly, as staff and funds are prioritised to more commercial programmes. Under the post-2000 partnering model, the same companies can still restrict costs and risks but in a far more productive way, focussing on discovery of breakthrough leads in the knowledge that others are available to help develop these and deliver them to patients.

## What Does This Mean for Government Policies?

There is a clear disjunct between the reality of neglected-disease activity and current government thinking, which is focussed on “commercialising R&D to bring big companies back into the field”. This thinking is built firmly on the beliefs outlined at the start of this report and is now significantly out of kilter with the industry neglected-disease drug landscape.

Two policy issues stand out. Firstly, there is an urgent need to support the new model of neglected-disease drug development, in particular the PPP approach, which is already generating new drugs, is highly cost-effective, appears to offer the highest health value, and is a crucial factor in continuing cost-effective industry involvement in neglected-disease R&D. On this point, we note—and welcome—the recent G8 commitment to “increasing direct investment … through such mechanisms as Public Private Partnerships … to encourage the development of … drugs for AIDS, malaria, TB and other neglected diseases” [7]. We look forward to seeing the shape of new policies and mechanisms to make this commitment concrete, and encourage policy-makers to ensure that these are designed to incentivise optimal practices within the PPP approach, and to do so in the most cost-effective manner. Simply handing over cash may not be the best way.

Secondly, we suggest that policy-makers review their approach to “commercialising” R&D in light of the information above. If big companies tell us that public “commercial” markets are not a catalysing factor in their decision to engage in neglected-disease R&D, then we need to listen carefully to them. Policies to stimulate new multinational company activity are one thing; policies that shift existing industry activity from a not-for-profit approach to a for-profit approach are quite another, and may do so at a potential cost of many billions of dollars across all neglected-disease products. Policy-makers may also want to consider whether commercial incentives should be preferentially targeted toward smaller companies that have a closer fit with commercial neglected-disease markets, and whether these new incentives should be tailored to encourage industry-alone R&D, or to encourage partnered models, which metrics suggest may deliver a better health outcome. The latter is particularly a concern given the relative inexperience of most Western pharmaceutical companies (and in particular small companies) in later-stage clinical development and implementation of tropical disease or TB drugs for use in rural Africa or South Asia, as opposed to their undoubted experience in developing drugs for large-scale US and European disease markets.

## Next Steps

The post-2000 renewal of neglected-disease R&D activity is good news for patients with neglected diseases, but it is only a beginning. We hope that this closer analysis will contribute to our store of information, and allow development of policies to encourage and improve these promising new trends in neglected-disease drug development.

## Abbreviations

PPP: public-private partnership
TB: tuberculosis
TDR: Special Programme for Research and Training in Tropical Diseases
