# Correction: A Breakthrough in R&D for Neglected Diseases: New Ways to Get the Drugs We Need

**DOI:** 10.1371/journal.pmed.0020376

**Published:** 2005-10-25

**Authors:** Mary Moran

In *PLoS Medicine*, volume 2, issue 9, DOI: 10.1371/journal.pmed.0020302

In the third paragraph of the section “Neglected-Disease R&D Activity,” the sentence “Nearly one-third of these projects are at the clinical trial stage, including seven drugs now in Phase III trials…” should read instead “Nearly one-third of these projects are at the clinical trial stage, including six drugs now in Phase III trials….”

